# Developing Multi-Copy Chromosomal Integration Strategies for Heterologous Biosynthesis of Caffeic Acid in *Saccharomyces cerevisiae*

**DOI:** 10.3389/fmicb.2022.851706

**Published:** 2022-03-01

**Authors:** Hang Qi, Long Yu, Yuanzi Li, Miao Cai, Jiaze He, Jiayu Liu, Luyao Hao, Haijin Xu, Mingqiang Qiao

**Affiliations:** ^1^The Key Laboratory of Molecular Microbiology and Technology, Ministry of Education, College of Life Sciences, Nankai University, Tianjin, China; ^2^School of Light Industry, Beijing Technology and Business University, Beijing, China

**Keywords:** caffeic acid, *p*-coumaric acid, *Saccharomyces cerevisiae*, heterologous biosynthesis, multi-copy integration

## Abstract

Caffeic acid, a plant-sourced phenolic compound, has a variety of biological activities, such as antioxidant and antimicrobial properties. The caffeic acid biosynthetic pathway was initially constructed in *S. cerevisiae*, using codon-optimized *TAL* (*coTAL*, encoding tyrosine ammonia lyase) from *Rhodobacter capsulatus*, *coC3H* (encoding *p*-coumaric acid 3-hydroxylase) and *coCPR1* (encoding cytochrome P450 reductase 1) from *Arabidopsis thaliana* in 2 μ multi-copy plasmids to produce caffeic acid from glucose. Then, integrated expression of *coTAL via* delta integration with the *POT1* gene (encoding triose phosphate isomerase) as selection marker and episomal expression of *coC3H*, *coCPR1* using the episomal plasmid pLC-c3 were combined, and caffeic acid production was proved to be improved. Next, the delta and rDNA multi-copy integration methods were applied to integrate the genes *coC3H* and *coCPR1* into the chromosome of high *p*-coumaric acid yielding strain QT3-20. The strain D9 constructed *via* delta integration outperformed the other strains, leading to 50-fold increased caffeic acid production in optimized rich media compared with the initial construct. The intercomparison between three alternative multi-copy strategies for *de novo* synthesis of caffeic acid in *S. cerevisiae* suggested that delta-integration was effective in improving caffeic acid productivity, providing a promising strategy for the production of valuable bio-based chemicals in recombinant *S. cerevisiae*.

## Introduction

Caffeic acid (3,4-dihydroxycinnamic acid) is a natural phenolic compound, widely occurring in plants, and the precursor of other natural products, such as chlorogenic acid, rosmarinic acid and caffeic acid phenethyl ester (Wu et al., 2011; David et al., 2015). Moreover, caffeic acid has numerous applications in functional foods, natural health products and medicine, because of its biological properties, which include anti-oxidant (Son and Lewis, 2002), anti-virus (Ikeda et al., 2011), anti-cancer (Espindola et al., 2019), and anti-inflammatory activities (Chao et al., 2009). Although caffeic acid can be produced either by extraction from plants or by chemical synthesis, both approaches have drawbacks, such as complex separation processes, the production of organic wastes and high cost (Yoshimoto et al., 2005). With the development of synthetic biology and metabolic engineering, heterologous biosynthesis of caffeic acid in microorganisms (e.g., *Escherichia coli*, *Saccharomyces cerevisiae*) provides a potential alternative source (Cao et al., 2020).

**FIGURE 1:**
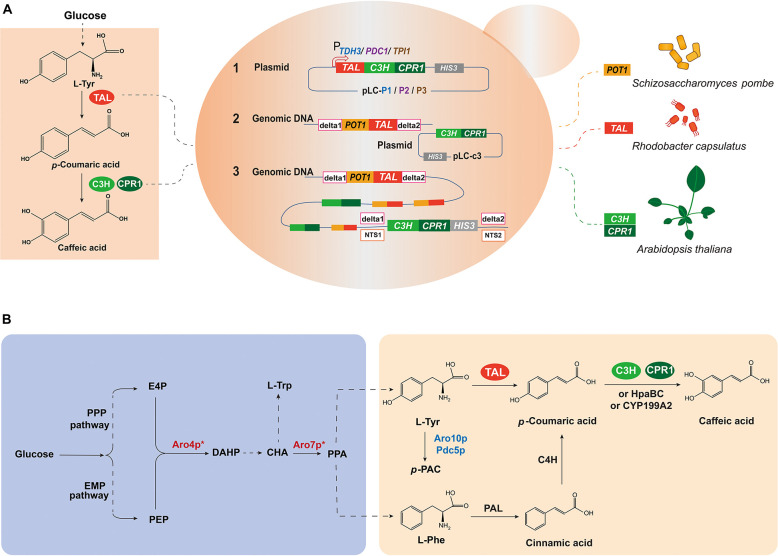
Design and construction of caffeic acid biosynthetic pathway from glucose in *S. cerevisiae*. **(A)** Schematic representation of the strategies used to construct the caffeic acid biosynthetic pathway. **(B)** Schematic diagram of the caffeic acid biosynthetic pathway. There is no downstream caffeic acid pathway from tyrosine in wild-type *S. cerevisiae*, so caffeic acid biosynthesis requires the introduction of heterologous genes. PPP pathway, pentose phosphate pathway; EMP pathway, Embden-Meyerhof-Parnas pathway; E4P, erythrose 4-phosphate; PEP, phosphoenolpyruvate; DAHP, 3-deoxy-D-arabinoheptulosonic acid-7-phosphate; CHA, chorismic acid; PPA, prephenate; HPP, para-hydroxyphenylpyruvate; L-Trp, L-tryptophan; L-Phe, L-phenylalanine; L-Tyr, L-tyrosine; *p*-PAC, para-hydroxy-acetaldehyde; Aro4p, DAHP synthase; Aro7p, chorismite mutase; Aro10p, phenylpyruvate decarboxylase; Pdc5p, pyruvate decarboxylase; TAL, tyrosine ammonia lyase; PAL, phenylalanine ammonia lyase; C4H, cinnamate-4-hydroxylase; C3H, *p*-coumarate 3-hydroxylase; CPR1, cytochrome P450 reductase 1; HpaBC, 4HPA3H, 4-hydroxyphenylacetate 3-hydroxylase; CYP199A2, cytochrome P450 monooxygenases. Enzymes in blue represent deletion, while in red represent overexpression, and * represents allosteric regulation.

As the precursors of caffeic acid in the plant phenylpropanoid pathway, aromatic amino acids (AAA; i.e., L-phenylalanine, L-tyrosine) can be produced by microorganisms through the shikimic acid pathway (Krivoruchko and Nielsen, 2015; Figure 1). There has been considerable research effort applied to relieving retro-inhibition and redirecting carbon flux from glucose into AAA metabolism to maximize the pool of L-tyrosine (Tyr) and L-phenylalanine (Phe) (Huccetogullari et al., 2019; Liu Q. et al., 2019). In plants, phenylalanine ammonia lyase (PAL) and two cytochrome P450 monooxygenases, cinnamate-4-hydroxylase (C4H) and *p*-coumarate 3-hydroxylase (C3H) participate in the synthesis of caffeic acid from L- Phe (Kim et al., 2011; Figure 1). Alternatively, *p*-coumaric acid (*p*-CA), the direct precursor of caffeic acid in plants, can be generated by microbial tyrosine ammonia lyase (TAL) from L-Tyr directly, instead of by C4H and PAL from L-Phe (Hernandez-Chavez et al., 2019). In addition, new enzymes in heterologous bacterial hosts have been investigated as alternatives for hydroxylation of *p*-CA to synthesize caffeic acid. Berner et al. (2006) introduced the C3H encoded by *sam5* from *Saccharothrix espanaensis* and Furuya et al. (2012) used the cytochrome P450 monooxygenases CYP199A2 from *Rhodopseudomonas palustris*. Lin and Yan (2012) found it is difficult to express the plant-originated P450 enzymes in prokaryotic systems, and identified that an *E. coli* endogenous non-P450 hydroxylase complex 4-hydroxyphenylacetate 3-hydroxylase (4HPA3H, also named HpaBC) was capable of converting *p*-CA to caffeic acid efficiently. Huang et al. (2013) reported the *de novo* production of caffeic acid (766.68 mg/L) by overexpressing the *E. coli* endogenous HpaBC. Jones et al. (2017) achieved the highest caffeic acid titer (1.03 g/L) from a simple carbon source using EcHpaBC combined with optimization of culture conditions.

*S. cerevisiae* is widely used as a host organism for the biosynthesis of plant-derived natural products and has GRAS (generally recognized as safe) status (Borodina and Nielsen, 2014). The first report on caffeic acid production (289.4 mg/L) in *S. cerevisiae*, using HpaB from *Pseudomonas aeruginosa* and HpaC from *Salmonella enterica* depended on supplementation with tyrosine (500 mg/L), the cost of which is too high for industrial production (Liu L. et al., 2019). Li et al. (2020) reported the *de novo* biosynthesis of caffeic acid (11.4 mg/L) from glucose, by simultaneous expression of RcTAL and C3H, assisted by cytochrome P450 reductase 1 (CPR1) in *S. cerevisiae*, without the need for precursor supplementation. In these two studies, they used the plasmid-based episomal expression method, at least two incompatible plasmids with diverse auxotrophic markers were applied to express caffeic acid. Recently, Zhou et al. (2021) reported the genomic integration of the caffeic acid synthetic genes *RgTAL* from *Rhodotorula glutinis*, *HpaB* from *P. aeruginosa* and *HpaC* from *S. enterica*, under the regulation of a modified *GAL* system and obtained the highest titer so far of caffeic acid (569.0 mg/L) in *S. cerevisiae*.

Compared with plasmid-based expression methods, multi-copy chromosomal integration offers advantages such as genetic regulation, passage stability, maintenance of target genes and less metabolic burden for the expression (Shi et al., 2014), and has not been applied for caffeic acid biosynthesis in yeast. Besides, in many cases, microbial heterologous synthesis of target products may be affected by a restricted copy number (Ko et al., 2020), so multi-copy integration (such as delta and rDNA integration) may be more suitable for heterologous synthesis (Malci et al., 2020) than single-copy site integration. In yeast, delta sites are located in the transposon Ty of the yeast genome and there are over 400 delta sites in *S. cerevisiae* (Lee and Da Silva, 1997). Genomic integration based on delta sites has been applied to the production of commercially valuable compounds by engineered *S. cerevisiae*, including resveratrol (Liu et al., 2017), glucaric acid (Chen et al., 2018), 2,3-butanediol (Yamada et al., 2017), and carotenoids (Yamada et al., 2018).

The ribosomal DNA (rDNA) sequence refers to the DNA sequence in the nucleus that encodes ribosomal RNA, which contains 100–140 repeat units in the *S. cerevisiae* XII chromosome (Kobayashi and Ganley, 2005). Each rDNA unit consists of two transcriptional regions (5S- and 35S-rDNA) and two non-transcribed spacers (NTS1 and NTS2) (Kobayashi et al., 2004). There have been many reports on the synthesis of secondary metabolic products using rDNA sites in *S. cerevisiae*, such as ginsenosides (Dai et al., 2013), glycyrrhetinic acid (Zhu et al., 2018), lycopene (Li et al., 2019), and (2S)-naringenin (Li et al., 2021). Thus, the multi-copy integration of heterologous pathway genes into *S. cerevisiae* may have great potential for the production of AAA-derived compounds like caffeic acid.

Previous studies have shown that deletion of the *TPI1* gene encoding triose phosphate isomerase from glycolytic pathway adversely impacts yeast cell growth with glucose as the sole carbon source (Compagno et al., 1999, 2001). Recently, Zeng et al. (2021) increased the endogenous 2 μ plasmid viability and plasmid copy number by moving the essential gene *TPI1* from chromosome to 2 μ plasmid, which provided an improved expression system in yeast. *POT1*, an essential gene from *Schizosaccharomyces pombe*, also encodes triose phosphate isomerase, which may struggle to function well in foreign host cells such as *S. cerevisiae* and therefore necessitate higher copy numbers in integration sites to compensate for the defective *TPI1* function (Hou et al., 2012). Thus, *POT1* could be used as a selection marker, making it possible to select recombinant *S. cerevisiae* integrated strains with higher gene copy number and stable production of heterologous products in non-selective medium such as YPD.

Herein, multi-copy integration expression strategies were applied and compared in producing caffeic acid heterologously from glucose using *S. cerevisiae*, providing fundamental insights to facilitate future research in this area.

## Materials and Methods

### Strains and Culture Conditions

Wild-type *S. cerevisiae* strain BY4742 and an engineered strain NKC6 were grown in synthetic complete (SC) medium: 1.7 g/L yeast nitrogen base (YNB), 20 g/L glucose, 5 g/L (NH4)_2_SO_4_ and 1.3 g/L amino acid mixture. Strain NKC6 was modified from wild-type *S. cerevisiae* strain BY4742 as described previously (Li et al., 2020), to increase the flux of tyrosine synthesis, by eliminating tyrosine-induced feedback inhibition and blocking the pathway flux to aromatic alcohols (Figure 1B). SC dropout medium without histidine (Sc-His) was used to screen and cultivate transformants: 20 g/L glucose, 1.3 g/L basic amino acid mixture without histidine, 5 g/L (NH4)_2_SO_4_, and 1.7 g/L YNB. Strain QK1 is a *TPI1* gene knockout yeast and needs to be cultured in modified synthetic complete ethanol (MSCE) medium: 1.7 g/L YNB, 0.5 g/L glucose, 5 g/L (NH4)_2_SO_4_, 1.3 g/L amino acid mixture and 10 mL/L ethanol. Strain QT3-20 was cultured in YPD medium: 20 g/L tryptone, 10 g/L yeast extract, 20 g/L glucose. For optimizing the fermentation medium, 4% glucose YPD medium (MYPD medium) was used: 20 g/L tryptone, 10 g/L yeast extract, and 40 g/L glucose. *E. coli* strain DH5α, used to construct the recombinant plasmids, was cultured in Luria-Bertani (LB) medium, containing 100 mg/L ampicillin.

### Plasmid Construction and Yeast Transformation

To construct episomal plasmids, the key constitutive strong enzyme promoters in the glycolytic pathway were selected to increase and stabilize the expression of exogenous genes. The fragments of constitutive promoters *TDH3*, *PDC1*, *TPI1*, and terminator *CYC1* were amplified from the *S. cerevisiae* genome using primers TDH-F/R, TPI-F/R, PDC-F/R, and CYC-F/R, respectively, and the gene *coTAL* was amplified from pLC-c1 using primers TAL-F/R. These DNA fragments were assembled to construct P*_TPI1_*_/_*_*TDH*3_*_/_*_*PDC*1_*-*coTAL*-T*_*CYC*1_* by overlap extension PCR (OE-PCR) and were cloned into pLC-c3 plasmid (*HIS3* auxotrophic marker) digested by *Sma*I and *Sac*II, respectively, resulting in the pLC-P1, pLC-P2, and pLC-P3 vectors. Then, pLC-P1, pLC-P2, and pLC-P3 vectors were transformed into BY4742 and NKC6, respectively, with lithium acetate and spread on SC-His plates for selection of successful transformants. Yeast colony PCR was performed for verification and NKP1, NKP2, NKP3, NKP4, NKP5, and NKP6 were obtained. In addition, the pLC-c3 vector was transformed into QT3-20, and NKP7 was obtained. The relevant plasmids and strains used are shown in Tables 1, 2 and the primer information is in Supplementary Table 1.

**TABLE 1:** Plasmids used in this study.

Plasmids	Description	Source
pSP-G1	2 μ ori, *URA3*, P*_*TEF*1_*-T*_*ADH*1_*, P*_*PGK*1_*-T*_*CYC*1_*, Amp*^r^*	Lab stock
pLC41	2 μ ori, *HIS3*, P*_*TEF*1_*-T*_*ADH*1_*, P*_*PGK*1_*-T*_*CYC*1_*, Amp*^r^*	Lab stock
pLC-c1	pSP-G1: P*_*PGK*1_*-*coTAL*-T*_*CYC*1_*, P*_*TEF*1_*-T*_*ADH*1_*	Lab stock
pLC-c3	pLC41: P*_*PGK*1_*-*coC3H*-T*_*CYC*1_*, P*_*TEF*1_*-*coCPR1*-T*_*ADH*1_*	Lab stock
pLC-P1	pLC41:P*_*PGK*1_*-*coC3H*-T*_*CYC*1_*-P*_*TEF*1_*-*coCPR1*-T*_*ADH*1_*-P*_*TDH*3_*-*coTAL*-T*_*CYC*1_*	This study
pLC-P2	pLC41:P*_*PGK*1_*-*coC3H*-T*_*CYC*1_*-P*_*TEF*1_*-*coCPR1*-T*_*ADH*1_*-P*_*PDC*1_*-*coTAL*-T*_*CYC*1_*	This study
pLC-P3	pLC41:P*_*PGK*1_*-*coC3H*-T*_*CYC*1_*-P*_*TEF*1_*-*coCPR1*-T*_*ADH*1_*-P*_*TPI*1_*-*coTAL*-T*_*CYC*1_*	This study
pLC-QTAL	pUC19 with *ALG9*, *coTAL* genes used for qPCR standard curve analysis	This study
pLC-QC3H	pUC19 with *ALG9, coC3H* genes used for qPCR standard curve analysis	This study
pLC-QCPR1	pUC19 with *ALG9, coCPR1* genes used for qPCR standard curve analysis	This study

**TABLE 2:** Strains used in this study.

Strains	Description	Source
*Saccharomyces cerevisiae* BY4742	*MAT*α, *ura3*Δ0, *leu2*Δ0, *his3*Δ1, *lys2*Δ0	EUROSCARF, Frankfurt, Germany
NKC6	BY4742; *aro10*Δ:*loxp*-P*_*PGK*1_*- *ARO7*^fbr^**-T*_*CYC*1_*-*loxp*;*pdc5*Δ:*loxp*-P*_*TEF*1_*-*ARO4*^fbr^**-T*_*ADH*1_*-*loxp*	Lab stock
NKP1	BY4742; pLC- P1	This study
NKP2	BY4742; pLC- P2	This study
NKP3	BY4742; pLC- P3	This study
NKP4	NKC6; pLC- P1	This study
NKP5	NKC6; pLC- P2	This study
NKP6	NKC6; pLC- P3	This study
NKP7	QT3-20; pLC-c3	This study
QK1	NKC6; *tpi1*Δ	This study
QT3-20	QK1 strain with delta1-P*_*TPI*1_*-*coTAL*-T*_*CYC*1_* -*POT1-*delta2-integrated	This study
D1-D9	QT3-20 strain with random multiple integration of P*_*PGK*1_*-*coC3H*-T*_*CYC*1_*-P*_*TEF*1_*-*coCPR1*-T*_*ADH*1_-HIS3* at delta sites	This study
N1-N15	QT3-20 strain with random multiple integration of P*_*PGK*1_*-*coC3H*-T*_*CYC*1_*-P*_*TEF*1_*-*coCPR1*-T*_*ADH*1_-HIS3* at NTS sites	This study

### Construction of Integrated Expression Strains

The primers 2C-F1/R1, 2C-F2/R1 were used to clone the *coC3H* and *coCPR1* gene cassettes from pLC-c3. The primers HIS-F1/R1, HIS-F1/R2 were used to clone *HIS3* from pLC41. The primers de1-F/R and de2-F/R were used to clone delta1 and delta2 from *S. cerevisiae* genomic DNA. The primers NTS1-F/R and NTS2-F/R were used to clone NTS1 and NTS2 from *S. cerevisiae* genomic DNA. The delta1, *coC3H*, *coCPR1*, *HIS3*, and delta2 fragments were assembled by OE-PCR with primers de1-F and de2-R. The NTS1, *coC3H*, *coCPR1*, *HIS3*, and NTS2 fragments were assembled with primers NTS1-F and NTS2-R. Two types of purified DNA fragments were transformed, respectively, into *S. cerevisiae* QT3-20 in equimolar amounts, with the LiAc method. Information on strains used in this study is summarized in Table 2.

### Strain Growth Curve and Passage Stability Analysis

A single colony of each strain was selected and inoculated in corresponding liquid medium (5 mL) to the logarithmic phase, at 30°C and 220 rpm. The seed cultures of three parallel transformants were inoculated to an OD_600_ of 0.1 in SC-His (or YPD, MYPD) liquid medium, in three 20 mL shake flasks. The cells were cultured at 30°C with orbital shaking at 220 rpm for 120 h (or 168 h). Samples were collected every 12 h for OD_600_ measurements and product determination.

The passage stability analyses of D9 and N15 were carried out to determine the stability of production and gene copy number. The primary strains were grown in YPD medium with an initial OD_600_ of 0.1, then transferred to fresh medium when the cells reached the late logarithmic phase, for 100 passages, with samples taken from the 50th and 100th generations. The samples from the primary, 50th and 100th generations were streaked and stored on solid plates, then the growth curves, gene copy numbers, and titers of products were determined for passage stability analyses.

### Determination of Gene Copy Number, Gene Expression Level and the Total Protein Concentration

To quantify gene copy number, the Ct values of target and reference genes were compared (Shi et al., 2014). Plasmid pLC-QTAL (pLC-QC3H/pLC-QCPR1), containing one copy each of *coTAL* (*coC3H*/*coCPR1*) and *ALG9* (encoding a mannosyl-transferase), was adopted as template for *coTAL* (*coC3H*/*coCPR1*/*ALG9*) quantitative polymerase chain reaction (qPCR) standard curve analysis. The reference *ALG9* gene was amplified using the primer QALG9-F/Q ALG9-R, whereas the target *coTAL* (*coC3H*, *coCPR1*) gene was amplified using the primer pair QTAL-F/QTAL-R (QC3H-F/QC3H-R, QCPR1-F/QCPR1-R) (Supplementary Table 1). Genomic DNA of the recombinant *S. cerevisiae* strain (1 μL) was used as a template for qPCR analysis. Each sample was tested three times and three parallel reactions were set each time. The qPCR program was as follows: 95°C for 5 min, 40 cycles of 95°C for 10 s, 60°C for 30 s, and 30 s at 72°C.

RNA was extracted from the yeast cells in the late logarithmic stage with the same OD_600_ value using an RNA pure yeast kit (CWBIO, Beijing, China), and the cDNA was synthesized with a PrimeScriptRT RT Master Mix reagent kit (TaKaRa, Tokyo, Japan). qPCR analysis was performed with SYBR Green qPCR Master Mix (Bimake, Houston, TX). Gene relative expression level was quantified by the comparative threshold cycle (2^–^*^ΔΔCT^*) method. All experiments were performed in triplicate.

The total protein concentration was determined by two methods. One is BCA kit according to instruction (CWBIO, Beijing, China), and the other is Kjeldahl nitrogen determination method through automatic elemental analyzer (Leeman, Rome, Italy).

### Analysis of Substrate and Products in Fermentation Media

To determine the concentration of *p*-CA and caffeic acid, the samples cultured in SC drop-out medium were centrifuged at 12,000 × *g* for 5 min. After filtration through 0.22 μm filters, the supernatants were analyzed by high-performance liquid chromatography (HPLC). For samples fermented in MYPD medium, supernatants were extracted with ethyl acetate and dried with a centrifugal vacuum concentrator, as described previously (Li et al., 2020). The *p*-CA and caffeic acid were quantified using a CoMetro 6000 HPLC instrument (CoMetro, South Plainfield, NJ, United States), equipped with an Inertsil ODS-3/C18 column (250 mm × 4.6 mm, 5 μm; GL Sciences, Tokyo, Japan) and a CoMetro 6000 UV detector. The gradient program was performed with solvent A (5:95 acetonitrile/water, with 0.1% v/v TFA) and solvent B (acetonitrile/0.1% TFA) as the mobile phases, at a flow rate of 1 mL/min, a detection wavelength of 310 nm and an injection volume of 10 μL. The concentrations of *p*-CA and caffeic acid were calculated by comparison with standard solutions of *p*-CA and caffeic acid in absolute ethanol.

## Results

### Construction of the Caffeic Acid Biosynthetic Pathway in *Saccharomyces cerevisiae*

**FIGURE 2:**
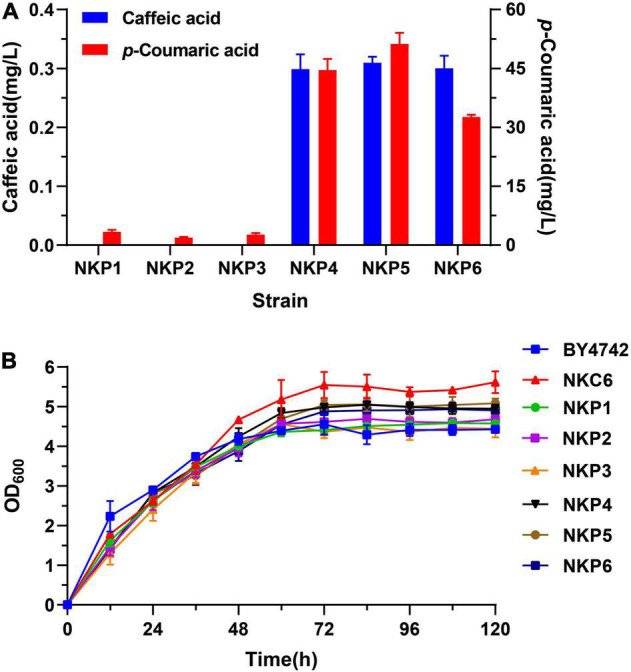
Expression of the caffeic acid synthesis pathway in *S. cerevisiae* transformed with the 2 μ plasmids in Sc-His medium, cultured by shake-flask fermentation for 120 h. (A) Production of *p*-coumaric acid and caffeic acid. (B) Growth curves of NKP1-NKP6 and the control strains BY4742 and NKC6. Average ± standard deviations were calculated from three biological replicates.

For heterologous biosynthesis of caffeic acid, codon-optimized *TAL* (*coTAL*) from *Rhodobacter capsulatus*, *coC3H* and *coCPR1* from *Arabidopsis thaliana* were selected, as reported previously (Li et al., 2020). Episomal expression strains were constructed by introducing the 2 μ multi-copy plasmids, pLC-P1, pLC-P2, or pLC-P3 (each harboring three gene cassettes) into wild-type *S. cerevisiae* BY4742 and a strain NKC6, optimized for the L-tyrosine biosynthetic pathway, which were designated as NKP1-6, respectively. NKP1-3 all produced a negligible amount of caffeic acid, and a small amount of *p*-CA (< 5 mg/L). While NKP4-6 all produced minute amounts of caffeic acid at about 0.3 mg/L and significantly higher production of *p*-CA. The growth rate of NKC6 was higher than the other strains, i.e., it was not affected by optimization of the L-tyrosine metabolic pathway, whereas the introduction of the caffeic acid hetero-synthesis pathway lowered the growth rates of NKP4-6, compared with NKC6 (Figure 2).

### Combination of Integrated Expression of Codon-Optimized *TAL* and Episomal Expression of *coC3H*, *coCPR1*

#### Enhancement of *p*-Coumaric Acid Supply by *POT1*-Mediated Delta Integration and Introduction of Episomal Plasmid

**FIGURE 3:**
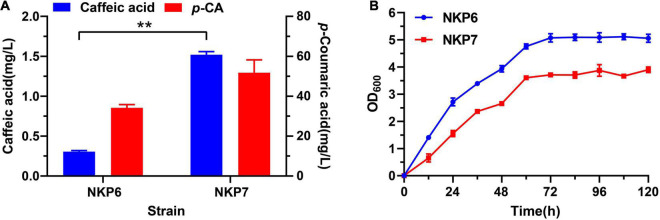
Comparison of caffeic acid production and growth curves of episomally expressed strains NKP6 and NKP7 after Sc-His shake-flask fermentation for 120 h. (A) Comparison of caffeic acid titers between NKP6 and NKP7. (B) Comparison of growth curves between NKP6 and NKP7. Averages ± standard deviations were calculated from three biological replicates (**P < 0.01).

The *coTAL* expression cassette was integrated into the genome of *TPI*-knockout strain QK1 using *POT1*-mediated delta integration strategy, and the selected recombinant strain QT3-20 with 19 copy *coTAL* integrated exhibits excellent genetic stability and stable production of *p*-CA during long-term fermentation in rich medium. Thus, we hypothesized strain QT3-20 could provide a stable *p*-CA supply for downstream products such as caffeic acid without the need of external precursor supplementation (Figure 1A). After shake-flask fermentation for 120 h in Sc-His medium, the caffeic acid titer of NKP7 (1.52 mg/L) harboring plasmid pLC-c3 was 4.1-fold that of NKP6 (0.3 mg/L) under the same fermentation conditions (Figure 3A). Expanding the *p*-CA supply significantly increased caffeic acid production, but the growth rate of NKP7 was significantly lower than that of NKP6 (Figure 3B), indicating plasmid instability and excessive metabolic burden imposed by plasmid DNA and foreign protein, which may cause metabolic, genetic and physiological changes, and reduced product yield from long-term fermentation (Collins et al., 2013).

#### Comparison Between Caffeic Acid-Producing Strains NKP4-7 in Rich Medium

**FIGURE 4:**
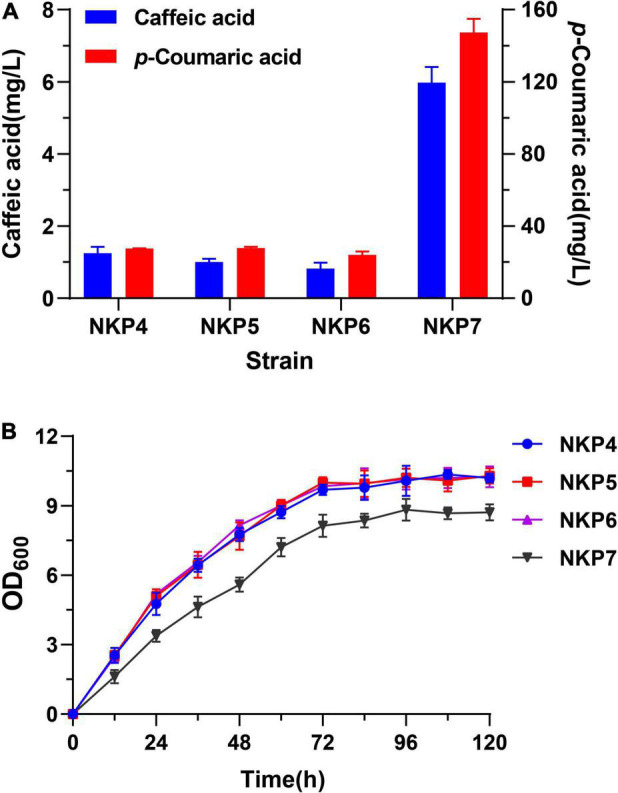
Performance of episomally expressed strains NKP4, NKP5, NKP6, and NKP7 in MYPD medium fermented for 120 h. (A) Production of *p*-CA and caffeic acid. (B) Growth curves. Average ± standard deviations were calculated from three biological replicates.

The episomal expression strains NKP4, NKP5, NKP6, and NKP7 which could produce caffeic acid successfully were fermented in MYPD medium for 120 h; final *p*-CA and caffeic acid production and growth curves were determined (Figure 4). These strains all produced more caffeic acid in MYPD medium than in Sc-His medium, with NKP7 producing a markedly higher titer of caffeic acid (5.98 mg/L) than the other strains (< 1.5 mg/L) and 2.93-fold higher than in Sc-His medium (1.52 mg/L; Figure 3A). Considering that there is no report about caffeic acid degradation by endogenous *S. cerevisiae* enzymes by far, the increased caffeic acid titers may result from more *p*-CA conversion. Besides, the *p*-CA titers of NKP4-6 were much lower in MYPD medium than in Sc-His medium (Figures 2A, 4A). In addition to higher biomass and more conversion to caffeic acid, the enriched MYPD medium may also endow strains with a larger number of *p*-CA decarboxylases. The high number of endogenous decarboxylases in *S. cerevisiae* such as phenylacrylic acid decarboxylase (PAD1) and ferulic acid decarboxylase (FDC1) have been shown to possess *p*-CA decarboxylase activity (Mukai et al., 2010; Koopman et al., 2012). And the decreased *p*-CA titers are in accordance with Combes et al. (2021) who have found the limitation of *p*-CA accumulation in fermentation broth and observed 4-vinylphenol as the main *p*-CA degradation product. Thus, apart from the conversion to caffeic acid, the decreased *p*-CA titers may also result from *p*-CA degradation being faster than its synthesis in rich medium.

### Optimized Caffeic Acid Biosynthesis by Two-Steps Multi-Copy Integration

#### Caffeic Acid Synthesis by Delta and Ribosomal DNA Integration Into Strain QT3-20

**FIGURE 5:**
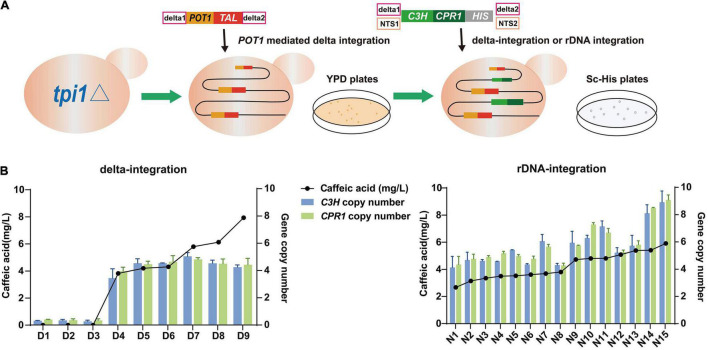
Construction of *p*-CA high-yielding strain QT-3-20 by *POT1*-mediated delta integration, then introduction of caffeic acid biosynthesis genes *coC3H* and *coCPR1*, by delta integration, or rDNA integration. (A) Strategy for construction of integrated expression strains. (B) Resulting copy numbers of *coC3H* and *coCPR1* genes and final caffeic acid production by delta- and rDNA-integrated strains, after fermentation in YPD medium for 120 h. Average ± standard deviations were calculated from three biological replicates.

To further multiplex the caffeic acid biosynthetic pathway into the *S. cerevisiae* QT3-20 genome, the *coC3H* and *coCPR1* cassettes, integration sites (delta or NTS) and the *HIS* selection marker, were ligated into two types of purified DNA fragments in equimolar amounts for integration (Figure 5A). 15 colonies were randomly selected from each of two Sc-His plates (Total clone No. 70 for rDNA-integration and 31 for delta-integration) and colony PCR was performed to verify whether the *coC3H* and *coCPR1* genes were successfully integrated into the genome. The integration efficiency of the *coC3H* and *coCPR1* genes at the genomic rDNA locus was 2.76-fold higher than that of delta integration (data not shown). The selected successful strains D1-D9 and N1-N15 were retained for subsequent analysis.

#### Comparison of Gene Copy Numbers Between Delta-Integrated Strains and Ribosomal DNA-Integrated Strains

To analyze the effects of the copy number of the genes *coC3H* and *coCPR1* on the biosynthesis of caffeic acid and compare the differences between delta and rDNA integration strategies, the 24 verified strains were fermented in YPD medium and subjected to qPCR and HPLC analyses (Figure 5B). The copy number of the *coC3H* and *coCPR1* genes in each strain was very similar and of the nine delta-integrated strains (D1-9), D9 had the highest caffeic acid production, at 7.9 mg/L, with *coC3H* and *coCPR1* copy numbers of 4.2 and 4.4, respectively. All of the 15 rDNA-integrated strains (N1-15) produced caffeic acid, the best performer being N15 (5.9 mg/L), with *coC3H* and *coCPR1* copy numbers of 8.9 and 9.1, respectively. Significant linear relationships were observed between caffeic acid production and the *coC3H* (or *coCPR1*) copy number, in both the delta- and rDNA-integrated strains (Supplementary Figure 1), indicating that a high copy number of the caffeic acid biosynthetic pathway genes increases caffeic acid production.

#### Comparison of Transcriptional Levels of Key Genes Between Delta-Integrated Strains and Ribosomal DNA-Integrated Strains

**FIGURE 6:**
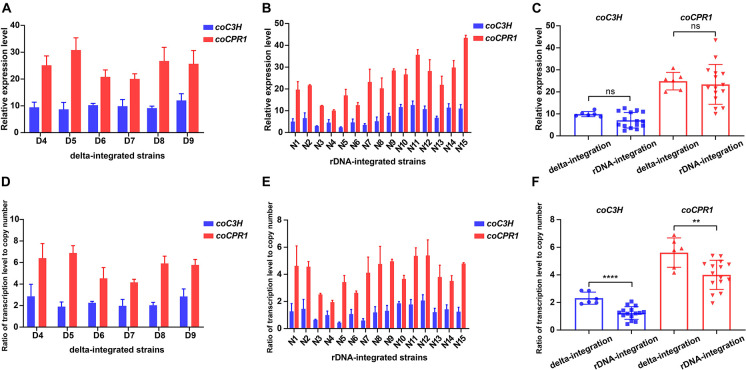
Transcription levels of the *coC3H* and *coCPR1* genes in delta- and rDNA-integrated strains that could produce caffeic acid. qPCR analysis of the expression levels of *coC3H* and *coCPR1* genes in delta integrated strains (A) and in rDNA integrated strains (B); comparison of *coC3H* and *coCPR1* transcription levels between delta- and rDNA-integrated strains (C); ratio of *coC3H* and *coCPR1* transcription levels to gene copy number in delta integrated strains (D) and in rDNA integrated strains (E); ratio of *coC3H* and *coCPR1* transcription levels to gene copy number between delta- and rDNA-integrated strains (F). Average ± standard deviations were calculated from three biological replicates. ** represents *p* < 0.01, **** represents *p* < 0.0001.

To compare the transcriptional levels of the genes *coC3H* and *coCPR1*, between the two types of integrated strains, strains that produced detectable caffeic acid were subjected to qPCR, which showed that the transcription level of *coCPR1* was generally higher than that of *coC3H*. For the delta-integrated strains, the transcription level of *coC3H* was 8.7–12, whereas that of *coCPR1* was 20–30.8 (Figure 6A). For the rDNA-integrated strains, the transcription level of *coC3H* was 2.4–12.6, whereas that of *coCPR1* was 10–43.5 (Figure 6B). There was no significant difference between the delta- and rDNA-integrated strains in terms of *coC3H* and *coCPR1* transcription levels (Figure 6C), but the ratio of overall transcription level to gene copy number of delta-integrated strains was significantly higher than that of rDNA-integrated strains (Figures 6D–F).

#### Total Protein Level and Passage Stability Analyses

In addition, the total protein level of these strains was determined by BCA colorimetric method and Kjeldahl method (Supplementary Figure 2). The results showed that there was no significant difference in the proportion of the wet weight (BCA colorimetry) or dry weight (Kjeldahl method) of the protein between the control strain NKC6 and engineered strain QT3-20 (or D9, N15) in the stable phase. And the stability of caffeic acid biosynthesis gene expression is essential for industrial production, so the passage stabilities of strains D9 and N15 were determined, showing that growth rates, gene copy numbers and production of *p*-CA and caffeic acid remained at their original level after subculturing in rich medium for 100 generations (Supplementary Figure 3). The growth curves, gene copy numbers and production levels of strains after subcultures were consistent with the primary strains, indicating the stability of the integrated genes into the delta and NTS sites.

#### Production of Caffeic Acid in Rich MYPD Medium

**FIGURE 7:**
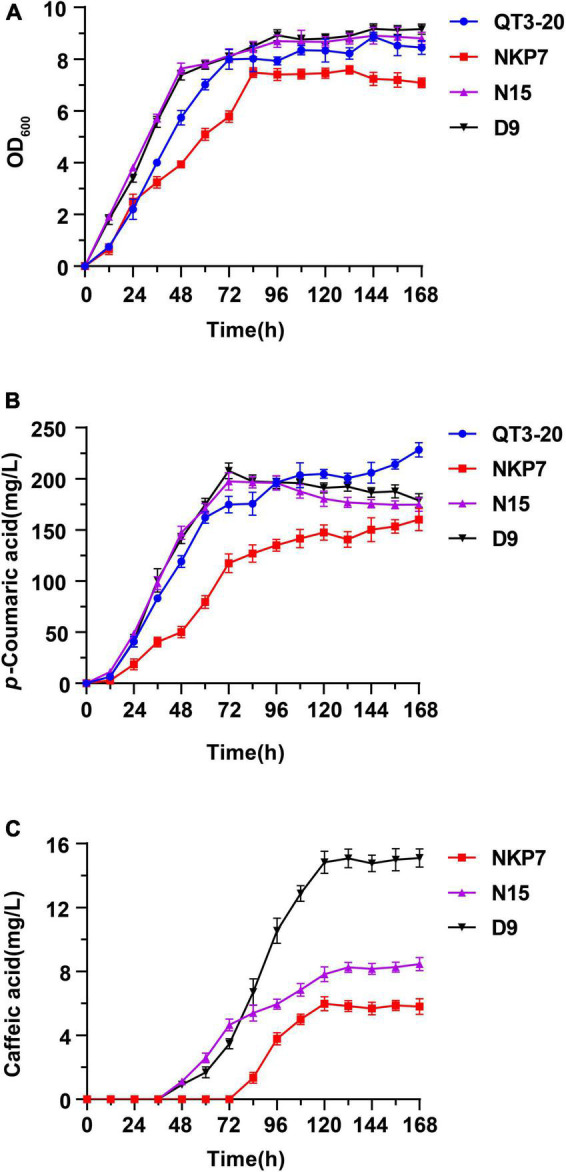
Time courses of engineered strains QT3-20, NKP7, N15, and D9, shake flask fermented in MYPD medium for 168 h: (A) Growth curves; (B) *p*-CA production; (C) Caffeic acid production. Average ± standard deviations were calculated from three biological replicates.

To enhance the growth rates and production of caffeic acid-producing strains from glucose, modified YPD medium containing 4% glucose (MYPD medium) was chosen to promote the synthesis of caffeic acid. The integrated expression strains N15 and D9, the starting strain QT3-20 and engineered strain NKP7, harboring pLC-c3 were cultured in MYPD medium for 168 h, for comparison of their time courses of growth, *p*-CA and caffeic acid production (Figure 7). D9 and N15 both entered the stationary phase earlier, at 48 h than QT3-20 (72 h) and NKP7 (84 h), which was similar in each case to the time when caffeic acid was first detected. The growth rate and final OD_600_ volume of NKP7 was much lower than that of the other three strains (Figure 7A).

There was a continuing upward trend in *p*-CA production during the fermentation by QT3-20 and NKP7 (Figure 7B), whereas that by N15 and D9 reached a peak at 72 h, then slightly decreased until the end of fermentation. The final *p*-CA titers of NKP7, N15 and D9 were 160, 174.7, and 178.7 mg/L, respectively, all lower than that of QT3-20 (228.4 mg/L). Caffeic acid production was first detected at 84 h in NKP7, much later than in N15 and D9 (48 h); the caffeic acid titers of all three strains increased until around 120 h, then plateaued (Figure 7C). The final caffeic acid titer of D9 was 15.1 mg/L, 1.8-fold that of N15 (8.5 mg/L) and 2.6-fold that of NKP7 (5.8 mg/L), under the same fermentation conditions.

## Discussion

In this study, the valuable product caffeic acid was heterologously synthesized through several strategies in *S. cerevisiae* (Figure 1), laying a foundation for further engineering of microbial cell factories. There was concern that using more than two incompatible plasmids from the same incompatibility group to carry genes expressing caffeic acid biosynthetic enzymes would lead to plasmid instability (Lindsey et al., 2011; Wang et al., 2021). Therefore, three gene cassettes were assembled into one plasmid skeleton to overcome plasmid incompatibility. Strains NKP4-6 outperformed NKP1-3, in terms of caffeic acid production, indicating that NKC6 provided more endogenous tyrosine for downstream heterologous synthesis of *p*-CA and caffeic acid, than the wild-type BY4742 strain, which is consistent with the report of Li et al. (2020) But the caffeic acid titers of NKP4-6 were still low (0.3 mg/L).

Huang et al. (2013) proved more *p*-CA supply would increase the production of caffeic acid. In the constructed caffeic acid biosynthesis pathway, *p*-CA serves as the direct precursor of caffeic acid. The supply of precursors is an important factor affecting the yield of products in microorganisms, but adding external precursors is likely to be problematic because exogenous compounds may adversely impact glucose metabolism (Du et al., 2011; Zhang and Hong, 2020). Thus, to be independent of external precursor supplementation, we enhanced the supply of precursor *p*-CA by employing high-copy *coTAL* integrated strain QT3-20, which can grow in YPD medium without selection pressure and provide stable *p*-CA precursor for the subsequent synthesis of caffeic acid. The caffeic acid titer of NKP7 haboring plasmid pLC-c3 was remarkably higher than that of NKP4-6 in both selective Sc-His and rich MYPD media, stating that expanding *p*-CA supply by QT3-20 did significantly increase caffeic acid production.

Previous studies showed that plasmid instability hinders the stable expression of exogenous genes and that expressing multiple biosynthetic pathway genes in a single copy usually cannot meet the need for high expression (Malci et al., 2020; Li et al., 2021). Therefore, multi-copy random chromosomal integration at multiple loci across the genome may be a superior alternative to produce high-yielding strains with optimal copy numbers of foreign genes (Da Silva and Srikrishnan, 2012). In this design, delta and rDNA clusters were selected as integration sites. Among the hundreds of potential integration sites, only a few copies of *coC3H* and *coCPR1* were integrated into the QT3-20 genome. In general, the copy numbers of *coC3H* and *coCPR1* from rDNA integration were higher than those from delta integration, possibly because the length of NTS sites being much longer than that of delta sites; and the use of longer homology arms improves homologous recombination frequency in *S. cerevisiae* (Storici et al., 2003; Maervoet et al., 2014; Juhas and Ajioka, 2016). In addition, cocktail integration and single step integration were also performed, but the results were not as good as the two-steps integration (data not shown), which may be because *coTAL* and *coC3H* encode enzymes with different functions and arrange sequentially in the caffeic acid synthetic pathway.

Recent research indicates that gene copy number influences transcription levels, thereby affecting the production efficiency of heterologous proteins (Maury et al., 2016). Although there was no significant difference in *coC3H* and *coCPR1* transcription levels between all delta and rDNA integrated strains, the ratio of overall transcription level to gene copy number of delta-integrated strains was significantly higher than that of rDNA-integrated strains, indicating that delta integration is superior to rDNA integration in expression efficiency for caffeic acid heterologous biosynthesis. In *S. cerevisiae*, delta sites are dispersed across different chromosomes, whereas rDNA clusters are only found in chromosome XII; the intergenic regions are responsible for maintaining the stability of the nucleolus and stabilizing the rDNA repeat numbers (Cahyani et al., 2015). The disruption of even a few rDNA repeats among the hundreds of copies can affect cell viability, and hinder growth and development, because the intergenic region NTS1 contains an origin of DNA replication (rARS), whereas NTS2 contains both a bidirectional RNA polymerase II-dependent promoter and a replication fork barrier (RFB) (Kwan et al., 2013; Kobayashi and Sasaki, 2017). To see whether it affects translation, the total protein levels of the controlled strains NKC6, QT3-20 and engineered strains N15, D9 were measured (Supplementary Figure 2). There was no significant difference in the proportion of the wet weight (BCA colorimetry) or dry weight (Kjeldahl method) of the protein between these strains in the stable phase, showing that occupying less than 10% integration sites didn’t affect the translation and growth of integrated strains.

Caffeic acid production by D9 increased 50-fold, from 0.3 mg/L to 15.1 mg/L, after optimization, compared with the initial 2 μ multi-copy plasmid construct. The final caffeic acid titer of D9 was also 32% higher than in a previous report, by Li et al. (2020) (11.4 mg/L). Cytochrome P450 monooxygenase C3H is the rate-limiting enzyme in the caffeic acid biosynthetic pathway, *coC3H* copy number can be further increased, while the limited integration efficiency of exogenous biosynthetic pathways into *S. cerevisiae* needs to be improved. Shi et al. (2016) introduced double-stranded breaks (DSBs) in the delta sequences using the CRISPR-Cas system and substantially improved integration efficiency, which provides a potential strategy to further improve caffeic acid titers. Besides, NAD(P)H was required for catalyzing hydroxylation reactions by cytochrome P450s as a cofactor (Jung et al., 2011; Bernhardt and Urlacher, 2014; Xu et al., 2019), thus playing an essential role in involving in the *p*-CA hydroxylation reaction by C3H. Therefore, increasing the availability of NAD(P)H may improve C3H expression and its enzymatic activity.

## Conclusion

In conclusion, multi-copy strategies were employed in this work to heterologously manufacture caffeic acid from glucose in *S. cerevisiae*, the growth, titer, gene copy number, transcription level, total protein level, and strain robustness of the engineered integrated strains were characterized carefully, resulting in a 50-fold increase in caffeic acid biosynthesis without altering the strain’s genetic stability. Through the intercomparison of multi-copy techniques, this study illustrates the usefulness of multi-copy chromosomal integration methods in caffeic acid production in *S. cerevisiae*, hoping to provide more reference and build a foundation for future development of heterologous biosynthesis.

## Data Availability Statement

The raw data supporting the conclusions of this article will be made available by the authors, without undue reservation.

## Author Contributions

HQ and YZL conceived and designed the experiments. HQ performed the experiments, wrote, and edited the manuscript. LY, MC, JZH, JYL, and LYH assisted in experiments. HJX and MQQ contributed materials and revised the manuscript. All authors read and approved the final manuscript.

## Conflict of Interest

The authors declare that the research was conducted in the absence of any commercial or financial relationships that could be construed as a potential conflict of interest.

## Publisher’s Note

All claims expressed in this article are solely those of the authors and do not necessarily represent those of their affiliated organizations, or those of the publisher, the editors and the reviewers. Any product that may be evaluated in this article, or claim that may be made by its manufacturer, is not guaranteed or endorsed by the publisher.

## Supplementary Material

Supplementary Figure 1 Relationship between *coC3H* and *coCPR1* copy numbers and caffeic acid production in delta integrated strains (A,B) and in rDNA integrated strains (C,D). The R^2^ value is the linear regression correlation coefficient. *P* < 0.01 represents significant correlation.

Supplementary Figure 2 Determination of total protein level of the controlled strains NKC6, QT3-20 and engineered strains N15, D9. (A) Growth curve. All strains were inoculated with 0.1 initial OD into parallel shake flasks (YPD, 20 mL), and shaken at 30°C for 220 r/min for 120 h. OD_600_ was measured every 12 h. (B) Protein content (Wet Weight/mg). The undried strains were weighed with the same OD value, then disrupted ultrasonically. Afterward, the total protein concentration of each strain was analyzed using BCA colorimetric method; (C) Protein content (Dry Weight/mg). The strains were dried, weighed with the same OD, and the N content of each strain was determined by the trace element analyzer, then the figures were converted into protein content according to the Kjeldahl method. Averages ± standard deviations were calculated from three biological replicates.

Supplementary Figure 3 Passage stability analysis of N15 and D9. Growth curves (A,D), gene copy number (B,E), and titers of *p*-CA and caffeic acid (C,F) after 0 generations (primary strains), after 50 generations and after 100 generations. Averages ± standard deviations were calculated from three biological replicates.
