# Supplementary material for: Developing Multi-Copy Chromosomal Integration Strategies for Heterologous Biosynthesis of Caffeic Acid in Saccharomyces cerevisiae
Source: Front Microbiol. 2022 Mar 1;13:851706. doi: 10.3389/fmicb.2022.851706 (PMC8923693; doi:10.3389/fmicb.2022.851706)
Supplement: Supplementary file 1 [file Table_1.doc]

**Table S1.** Primers used in this study.

| **Primers** | **Sequences (5'-3')** |
| --- | --- |
| **For cloning** |  |
| TDH-F | GCTGCATTAATGAATCGGCCAAAAAACGAATATATACTAG |
| TDH-R | CAGTCTGTGATTGCAAAGTCATTTTGTTTGTTTATGTGTGTTTAT |
| TPI-F | GCTGCATTAATGAATCGGCCAGATCTACGTATGGTCATTTCTT |
| TPI-R | CAGTCTGTGATTGCAAAGTCATTTTTAGTTTATGTATGTGTTTTTTG |
| PDC-F | GCTGCATTAATGAATCGGCCCAAGGCAGAAACTAACTTCT |
| PDC-R | CAGTCTGTGATTGCAAAGTCATTTTGATTGATTTGACTGTGTT |
| CYC-F | ATCCGCTCTAACCGAAAAG |
| CYC-R | GGCCGATTCATTAATGCAGC |
| TAL-F1 | ATAAACACACATAAACAAACAAAATGACTTTGCAATCACAGACTG |
| TAL-F2 | AACACAGTCAAATCAATCAAAATGACTTTGCAATCACAGACTG |
| TAL-F3 | CAAAAAACACATACATAAACTAAAAATGACTTTGCAATCACAGACTG |
| TAL-R | CAACTTCTGTTCCATGTCGAC |
| de1-F | TCGAGGAGAACTTCTAGTATATTC |
| de1-R | CCTTGCTTGAGAAGGTTTTGGGACGCTCGAAGCTGATGAGCAGGTGTTGTG |
| 2C-F1 | CTCAACAGACACAACACCTGCTCATCAGCTTCGAGCGTCCCAAAACC |
| 2C-R1 | ATATAGAGTGTACTAGAGGAGGCCAAGGGAGCGACCTCATGCTATAC |
| His-F1 | GCTTTCTCAGGTATAGCATGAGGTCGCTCCCTTGGCCTCCTCTAGTACACTC |
| His-R1 | GTAGATGATAGTTGATTTCTATTCCAACAGGTACCGGCCGCAAATTAAAG |
| de2-F | CTCGAAGGCTTTAATTTGCGGCCGGTACCTGTTGGAATAGAAATCAACTATC |
| de2-R | GATATAGGAATCCTCAAAATGG |
| NTS1-F | GGTTGCGGCCATATCTACCAGAAAGC |
| NTS1-R | CCTTGCTTGAGAAGGTTTTGGGACGCTCGAAGCGTTGCAAAGATGGGTTGAAAGAG |
| 2C-F2 | GCCCTTCTCTTTCAACCCATCTTTGCAACGCTTCGAGCGTCCCAAAACC |
| His-R2 | GTGAGGAAAAGTAGTTGGGAGGTACTTCGGTACCGGCCGCAAATTAAAG |
| NTS2-F | CTCGAAGGCTTTAATTTGCGGCCGGTACCGAAGTACCTCCCAACTACTTTTCC |
| NTS2-R | ATAGTTTAACGGAAACGCAGGTG |
| **For verification** |  |
| pTAL-F | TTGGACGGAGCATTGACTTT |
| pTAL-R | TCAATCCTGGAGAGACTGCC |
| pC3H-F | AAGTTCCCTCCAGGTCCATC |
| pC3H-R | AAGGCAACAATCTGAAGTCG |
| pCPR1-F | CTTCAGCTTTGTACGCATCA |
| pCPR1-R | ATTCCTTCTGAGCACCTTCC |
| **For RT-qPCR** |  |
| QALG9-F | ACGCTATCTGTCCACTGGGTC |
| QALG9-R | AACAGCAGTCGAATGCGGTTC |
| QTAL-F | GAGGGCTAACGCAACTCCTG |
| QTAL-R | TTGAATTTGTGACAATGGCAAC |
| QC3H-F | AATGGGCTATGGCTGAAATG |
| QC3H-R | TGTTGGAGGGTGCAACCTAA |
| QCPR1-F | TCACCGTCACCGTAAGTAGCA |
| QCPR1-R | CAAAGGCATTGTCTGAGGAG |
