# Supplementary figures and images for: Developing Multi-Copy Chromosomal Integration Strategies for Heterologous Biosynthesis of Caffeic Acid in Saccharomyces cerevisiae
Source: Front Microbiol. 2022 Mar 1;13:851706. doi: 10.3389/fmicb.2022.851706 (PMC8923693; doi:10.3389/fmicb.2022.851706)

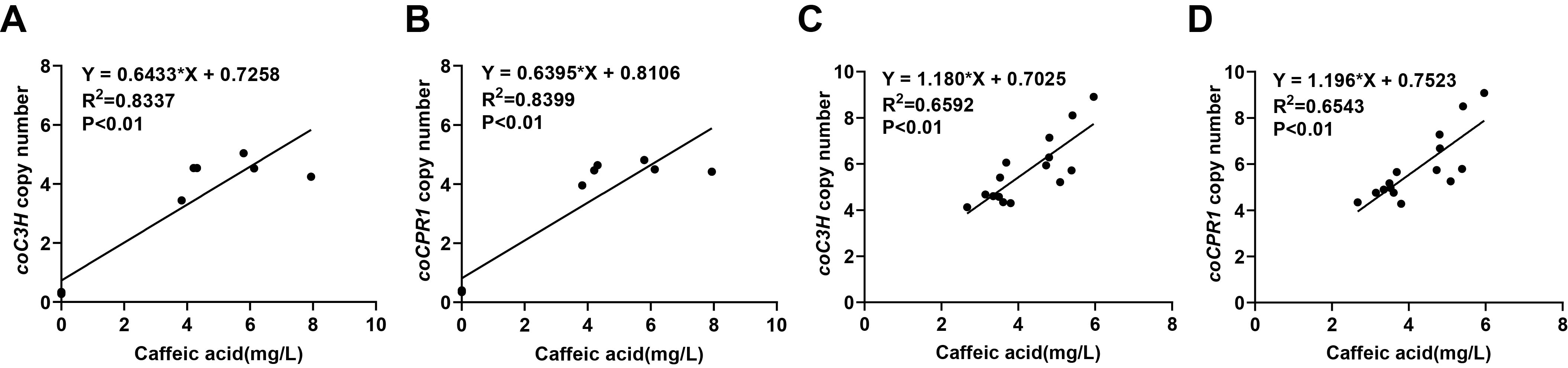

Supplement: Supplementary Figure 1 — Relationship between coC3H and coCPR1 copy numbers and caffeic acid production in delta integrated strains (A,B) and in rDNA integrated strains (C,D). The R2 value is the linear regression correlation coefficient. P < 0.01 represents significant correlation. [file Image_1.JPEG]

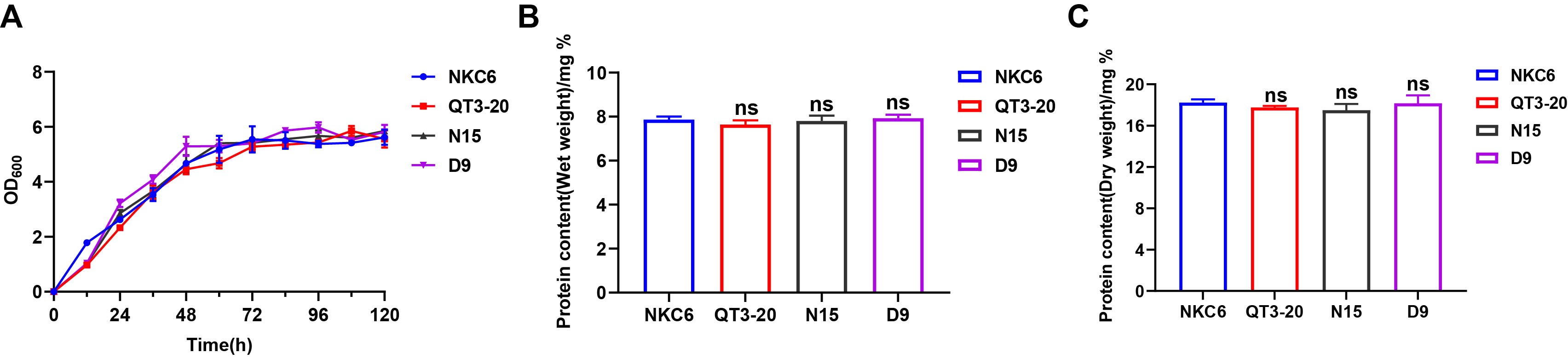

Supplement: Supplementary Figure 2 — Determination of total protein level of the controlled strains NKC6, QT3-20 and engineered strains N15, D9. (A) Growth curve. All strains were inoculated with 0.1 initial OD into parallel shake flasks (YPD, 20 mL), and shaken at 30°C for 220 r/min for 120 h. OD600 was measured every 12 h. (B) Protein content (Wet Weight/mg). The undried strains were weighed with the same OD value, then disrupted ultrasonically. Afterward, the total protein concentration of each strain was analyzed using BCA colorimetric method; (C) Protein content (Dry Weight/mg). The strains were dried, weighed with the same OD, and the N content of each strain was determined by the trace element analyzer, then the figures were converted into protein content according to the Kjeldahl method. Averages ± standard deviations were calculated from three biological replicates. [file Image_2.JPEG]

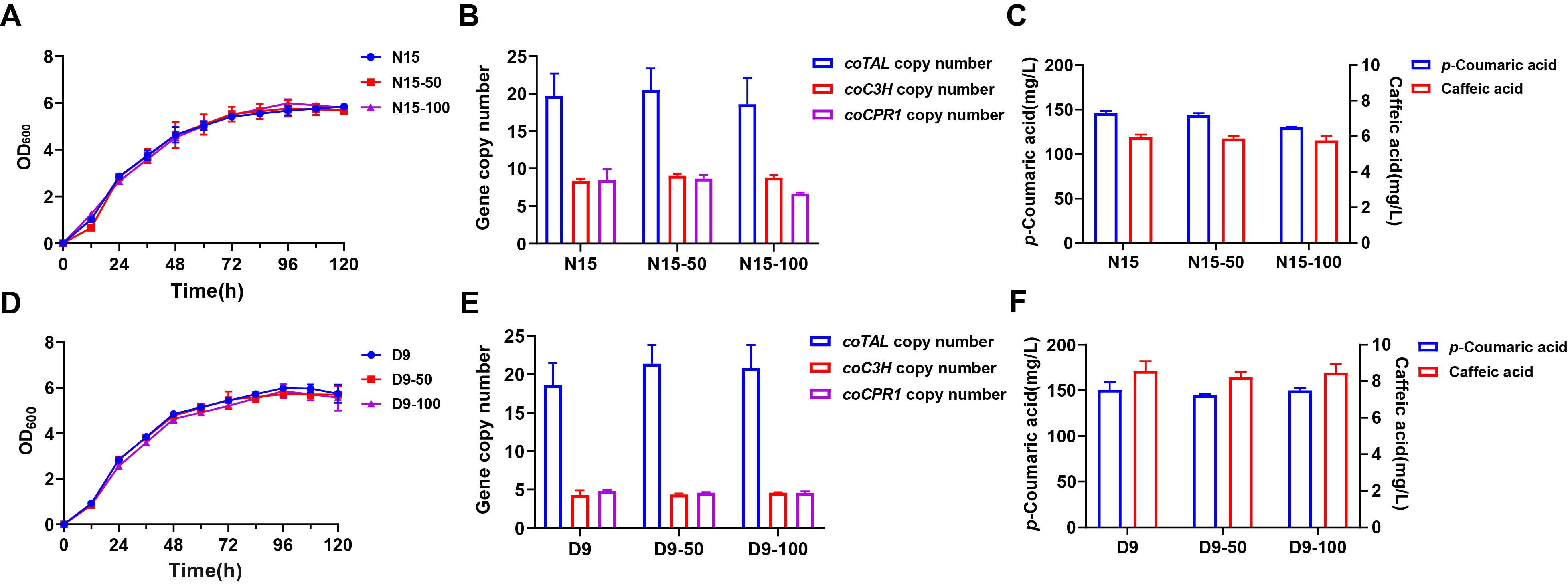

Supplement: Supplementary Figure 3 — Passage stability analysis of N15 and D9. Growth curves (A,D), gene copy number (B,E), and titers of p-CA and caffeic acid (C,F) after 0 generations (primary strains), after 50 generations and after 100 generations. Averages ± standard deviations were calculated from three biological replicates. [file Image_3.JPEG]
